# Promotion or remission: a role of noncoding RNAs in colorectal cancer resistance to anti-EGFR therapy

**DOI:** 10.1186/s12964-022-00960-x

**Published:** 2022-09-21

**Authors:** Shanshan Wei, Wenwei Hu, Jun Feng, Yiting Geng

**Affiliations:** 1 Department of Oncology, The Third Affiliated Hospital of Soochow University, 185 Juqian Street, Changzhou, 213003 Jiangsu China; 2 Jiangsu Engineering Research Center for Tumor Immunotherapy, The Third Affiliated Hospital of Soochow University, Changzhou, China

**Keywords:** Anti-EGFR monoclonal antibody, Colorectal cancer, Resistance, ncRNA, lncRNA, miRNA, cicrRNA

## Abstract

Anti-epidermal-growth-factor-receptor (EGFR) monoclonal antibodies (mAbs) are of great significance for RAS and BRAF wild-type metastatic colorectal cancer (mCRC) patients. However, the generation of primary and secondary resistance to anti-EGFR mAbs has become an important factor restricting its efficacy. Recent studies have revealed that non-coding RNAs (ncRNAs), especially long non-coding RNAs (lncRNAs), microRNAs (miRNAs), and circular RNAs (circRNAs), are implicated in anti-EGFR antibodies resistance, affecting the sensitivity of CRC cells to Cetuximab and Panitumumab. This paper briefly reviewed the research advance of the expression, signaling network and functional mechanism of ncRNAs related to anti-EGFR mAbs resistance in CRC, as well as their relationship with clinical prognosis and the possibility of therapeutic targets. In addition, some ncRNAs that are involved in the regulation of signaling pathways or genes related to anti-EGFR resistance, but need to be further verified by resistance experiments were also included in this review, thereby providing more ideas and basis for ncRNAs as CRC prognostic markers and anti-EGFR therapy sensitizers.

**Video Abstract**

**Supplementary Information:**

The online version contains supplementary material available at 10.1186/s12964-022-00960-x.

## Background

Currently, as one of the most common malignant tumors, colorectal cancer (CRC) has caused a huge global health burden. In 2020, there were 1.93 million new cases and 935,173 deaths from CRC worldwide, ranking the 3rd and 2nd among all cancers, respectively [1]. Anti-EGFR monoclonal antibodies (mAbs), currently including Cetuximab and Panitumumab, combined with chemotherapy are the preferred treatment options for patients with RAS and BRAF wild-type mCRC [2]. When EGFR is in an inactive configuration, anti-EGFR mAbs block the ligand-induced EGFR tyrosine kinase activation by competitively binding to the extracellular domain, thereby inhibiting the downstream signaling. EGFR is a transmembrane tyrosine kinase receptor that triggers two major intracellular signaling pathways by binding to ligands, namely the RAS/RAF/MEK/MAPK and the PI3K/PTEN/AKT pathway, the former of which controls gene transcription, cell cycle progression and cell proliferation, the latter of which activates anti-apoptotic and pro-survival signaling cascades [3, 4]. However, primary and secondary resistance limits the application of anti-EGFR mAbs. Some patients have primary resistance to anti-EGFR mAbs, and about 70% of patients who are originally sensitive to anti-EGFR mAbs will develop secondary resistance after receiving anti-EGFR mAbs treatment for 3–12 months [5].

**Fig. 1:**
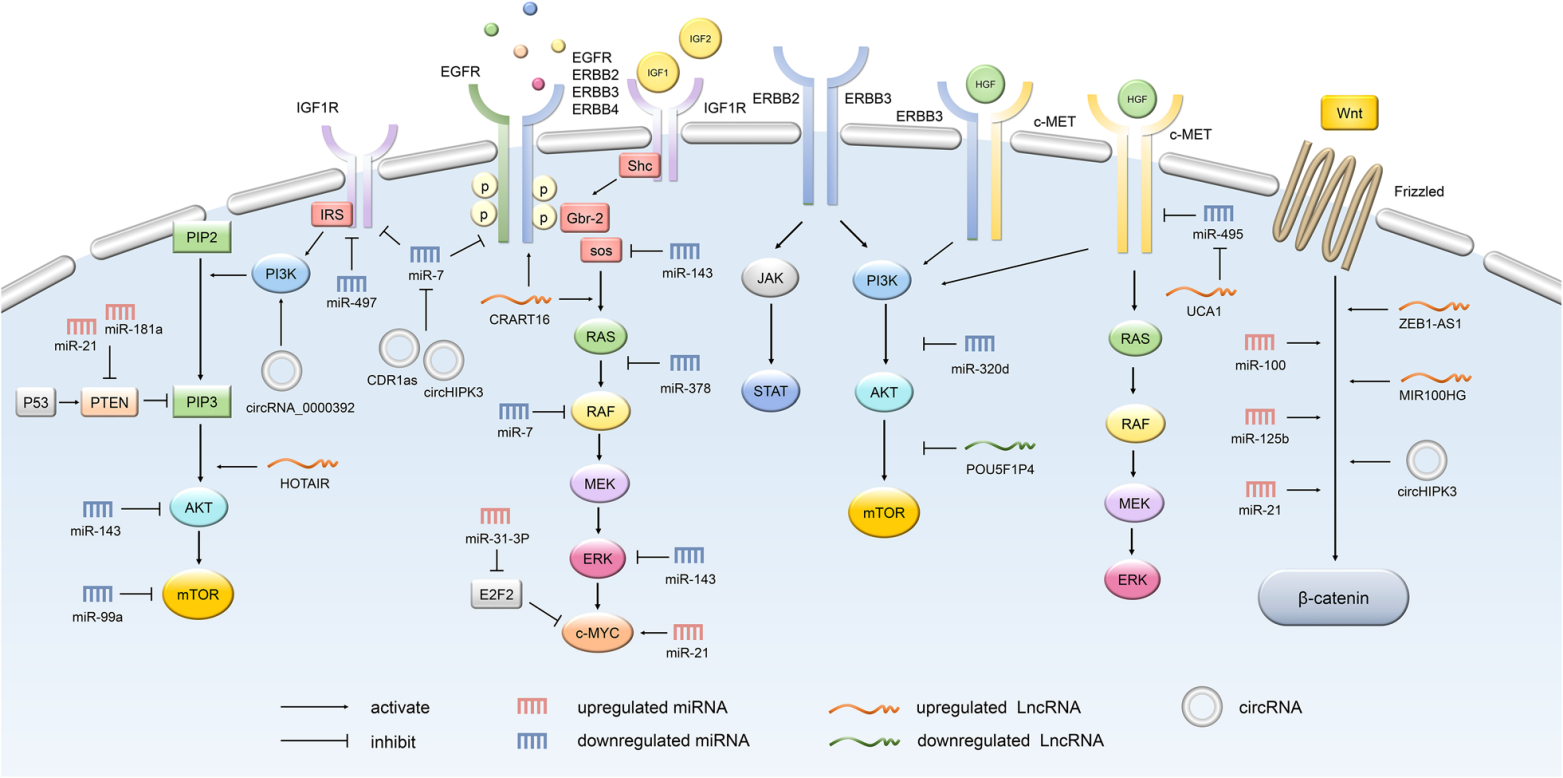
The main signaling pathways that affect the sensitivity to anti-EGFR therapy. The effect of ncRNAs on the sensitivity of anti-EGFR mAbs is largely mediated by intracellular signaling, including RAS/RAF/MEK/MAPK, RAS/RAF/MEK/ERK, PI3K/PTEN/ATK, c-MET/PI3K/PTEN/ATK, c-MET/RAS/RAF/MEK, IGFR/PI3K/PTEN/ATK, SCR/PI3K/PTEN/ATK, and JAK/STAT, etc. Some representative miRNAs, lncRNAs and miRNAs and their targets/pathways are shown in the figure

The genetic mechanisms of primary and acquired drug resistance to anti-EGFR mAbs have made great progress, including KRAS, NRAS, BRAF, PIK3CA and PTEN sequence mutations [6–10], as well as the amplification of MET and ERBB2 [11–13], also implicated in complex signaling pathways, including RAS/RAF/MEK/MAPK [7], RAS/RAF/MEK/ERK [7, 12], PI3K/PTEN/ATK [14], c-MET/PI3K/PTEN/ATK [15], c-MET/RAS/RAF/MEK [15], IGFR/PI3K/PTEN/ATK [16], SCR/PI3K/PTEN/ATK [17, 18], and JAK/STAT [19], etc. (Fig. 1). Among them, the status of RAS gene (wild/mutant) is most closely related to the efficacy of anti-EGFR mAbs. The CRYSTAL study, which is of great significance in CRC targeted therapy, found that the KRAS gene status has a predictive effect on the efficacy of mCRC patients, and is a powerful indicator to guide individualized treatment. In the overall survival (OS) analysis according to tumor gene mutation status, the efficacy of Cetuximab combined with FOLFIRI chemotherapy in KRAS wild-type patients was significantly better than that in the chemotherapy group, while in the KRAS mutant population there was no difference between the two treatment groups, even showing the opposite trend [20]. According to the re-understanding of RAS gene in CRYSTAL trial, on the basis of the stratified analysis of KRAS genes, the OPUS trial retrospectively screened 26 genes mutations (New RAS) with 4 additional KRAS codons (exons 3 and 4) and 6 NRAS codons (exons 2, 3 and 4). It was found that patients with any RAS mutation could not benefit from the combination of Cetuximab and FOLFOX4 chemotherapy [21]. In addition, about 5%-7% of mCRCs have BRAF V600E mutations [21], and BRAF-mutated mCRCs are less sensitive to chemotherapy and cannot benefit from anti-EGFR mAbs therapy [22]. After transiently reducing the phosphorylation level of ERK, a downstream effector of the MAPK signaling pathway, BRAF inhibitors rapidly feedback activated EGFR, and reactivated downstream signaling through the PI3K/AKT and CRAF pathways [23], which become the theoretical basis for the combined targeted therapy of BRAF inhibitors and Cetuximab. At present, anti-EGFR mAbs combined with BRAF inhibitors and MEK inhibitors have been recommended by major clinical guidelines such as the National Comprehensive Cancer Network (NCCN) for the standard treatment of BRAF V600E mutant mCRC. The other genes/signaling pathways mentioned above have also been found to be associated with anti-EGFR mAbs sensitivity, but lack of strong clinical trial evidence, so they have not yet become definite indicators for guiding anti-EGFR therapy in clinical practice. In contrast, studies on the non-genetic mechanisms of resistance to anti-EGFR mAbs are less reported.

**Fig. 2:**
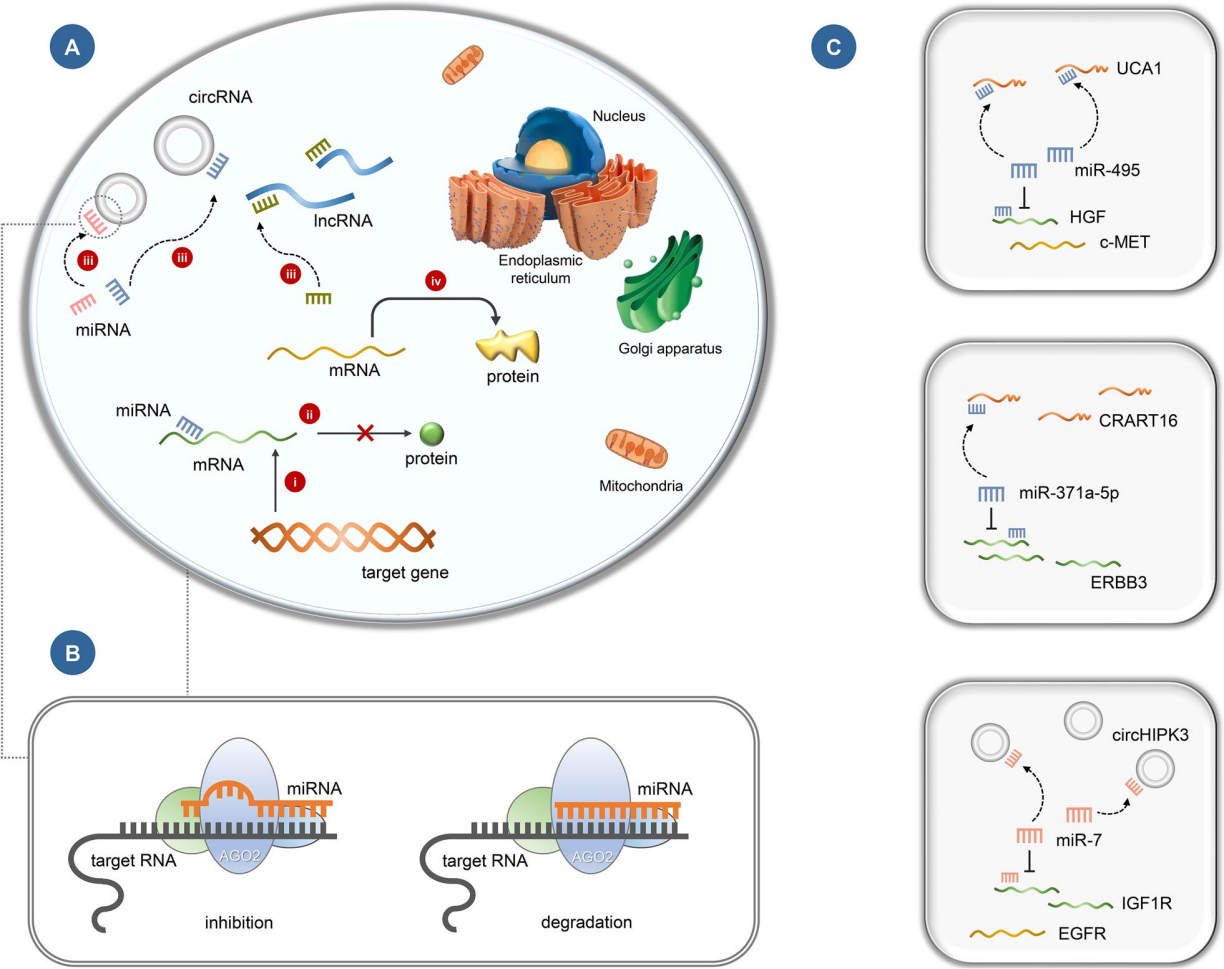
Mechanism of ceRNAs and ncRNAs involved in anti-EGFR resistance in CRC. **a** Mechanisms of lncRNAs and circRNAs as ceRNAs: (i) The target gene is transcribed into mRNA. (ii) The 3′-UTR of target mRNA is bound by and inhibited by miRNA, preventing protein translation. (iii) Free miRNAs in the cytoplasm are adsorbed by circRNAs and lncRNAs through miRNA response elements (MREs), resulting in a decrease in the level of miRNAs. (iv) The negative regulation of miRNA on target mRNA is weakened or eliminated, and mRNA is translated into protein, which mediates biological functions. **b** MREs are defined as regions on target RNAs, such as mRNAs, lncRNAs, and circRNAs, that can be bound by miRNAs through complementary base pairing. MREs are essentially short sequences, resulting in mRNAs degradation or translational inhibition. **c** ceRNA network associated with anti-EGFR mAbs sensitivity in CRC: UCA1/miR-495/HGF/c-MET, CRART16/miR-371a-5p/ERBB3, circHIPK3/miR-7/IGF1R/EGFR

Current studies have revealed that non-coding RNAs (ncRNAs), especially long non-coding RNAs (lncRNAs) and microRNAs (miRNAs), are important players in tumorigenesis and progression due to their effects on epigenetic regulation [24–27]. With the development of transcriptome analysis technology, the role of ncRNAs in the formation of anti-EGFR mAbs resistance has gradually attracted attention. NcRNAs account for 98% of the human genome and are RNA transcripts that do not encode proteins. According to their length, they can be divided into several types, including lncRNAs, miRNAs, and circRNAs. Other types include small nuclear RNAs (snRNAs), small nucleolar RNAs (snoRNAs), PIWI-interacting RNAs (piRNAs), small interfering RNAs (siRNAs), etc*.* [28]. There is a complex network of interactions and regulation among ncRNAs. Among them, lncRNAs and circRNAs can act as endogenous miRNA sponges, also known as competitive endogenous RNAs (ceRNAs), which are involved in the modulation of miRNAs at the post-transcriptional level [29, 30]. Specifically, miRNAs degrade or inhibit the expression of the target genes by recognizing and binding to the 3′ untranslated region (3′-UTR) of the target mRNAs. By competing with mRNAs for the binding sites of miRNAs, lncRNAs and circRNAs can derepress miRNAs from their target genes (Fig. 2A). In addition to lncRNAs and circRNAs, ceRNAs include endogenously transcribed pseudogenes [29, 31]. In recent years, ceRNAs have been found to be involved in tumor development, and ceRNA networks play an important role in resistance to anti-EGFR mAbs in CRC [30, 32, 33]. This paper mainly reviewed the lncRNAs, miRNAs, and circRNAs related to anti-EGFR mAbs resistance, and elucidated their correlation with anti-EGFR mAbs resistance for CRC and the potential mechanism, providing new methods and ideas for predicting the prognosis and treatment of CRC patients.

## MiRNAs and anti-EGFR therapy resistance

### Overview of miRNAs

MiRNAs are a class of single-stranded ncRNAs containing approximately 21 nucleotides that regulate gene expression at the post-transcriptional level [34, 35]. The biogenesis of miRNAs involves a series of tightly regulated bioprocessing steps. Primary miRNAs produced by miRNAs genes are spliced, nuclear exported and diced, and then exported from the nucleus to the cytoplasm to form mature double-stranded miRNAs. Mature and biologically active miRNAs combine with Argonaute (Ago) protein to form a miRNA-induced silencing complex (miRISC). After assembly, miRNAs can silence their mRNA targets by recognizing and binding to the 3′-UTR of the target mRNA, causing its degradation or translation inhibition [36]. The recognition of miRNA-mRNA is mainly performed by incomplete base pairing, involving a high degree of sequence complementarity between the 5′-end of the miRNA (the seed region) and the 3′-UTR of the mRNA [37, 38]. This incomplete base pairing and relatively short binding region (6–8 base pairs) allow a single miRNA to target and regulate multiple mRNAs implicated in a variety of biological processes, such as cell proliferation, motility, differentiation and apoptosis [39]. The abnormal expression, processing and function of miRNAs are implicated in almost all aspects of tumorigenesis and development [40]. According to their functions in the regulation of tumor-related pathways, miRNAs act as tumor suppressors or tumor promoters. Moreover, some miRNAs have been reported to be involved in tumor resistance, such as miR-125b [41] and miR-199a-5p [42].

### Upregulated miRNAs

MiR-100 and miR-125b, encoded by the polycistronic miRNA host gene MIR100HG intron, were upregulated in Cetuximab-resistant CRC cells [41]. Wnt/β-catenin signaling is considered to be a potent regulator of epithelial to mesenchymal transition (EMT) [43], which is involved in tumor metastasis and drug resistance [44]. DKK1, ZNRF3, RNF43, DKK3 and APC2 are negative regulators of the Wnt/β-catenin pathway. MiR-100 and miR-125b synergistically activate Wnt/β-catenin signaling by reducing the expression of these factors, thereby promoting Cetuximab resistance. The regulatory network between zinc finger transcription factors GATA6, MIR100HG and miR-125b underlies Cetuximab resistance. GATA6 inhibits MIR100HG expression, and miR-125b inhibits GATA6 expression, thereby enhancing MIR100HG expression [41]. MiR-125b, a member of the miR-125 family, plays a key role in many cancer-related processes, including cell proliferation, differentiation, and apoptosis. Currently, miR-125b has been recognized as an important regulator of human cancer. It is incorporated into the RNA induced silencing complex (RISC). In addition to its synergistic effect by binding to miRNAs from the same cluster, it can also lead to the target mRNAs degradation and inhibit the expression of lncRNAs such as MALAT1 [45]. Moreover, miR-125b can directly inhibit 20 new targets in the p53 network [46]. HER2 is a potential target of miR125b, and miR-125b may regulate the resistance to anti-EGFR mAbs partially by acting on HER2 [47].

### Downregulated miRNAs

MiR-7 is a tumor suppressor in many malignancies including CRC, and its expression is downregulated in CRC [48–50]. Detection of miR-7 levels in plasma is helpful in distinguishing CRC patients from healthy individuals, and thus can be considered as a potential biomarker [48]. The oncogenes YY1 and TYRO3 are direct targets of miR-7 and are involved in the regulation of p53, Wnt and PI3K/AKT/mTOR pathways, respectively. MiR-7 inhibits the proliferation, migration and invasion of CRC cells by inhibiting YY1 and TYRO3 [49, 50]. In addition, miR-7 regulates CRC cell proliferation and Cetuximab sensitivity by targeting EGFR and the downstream of RAS gene RAF-1 [51]. Furthermore, as a sponge of miR-7, cicrRNA CDR1as silencing can inhibit the expression of EGFR and insulin-like growth factor-1 receptor (IGF1R) [52].

MiR-143 and miR-145 are co-transcribed miRNAs originally found to be downregulated in CRC with tumor suppressive effects [53], and subsequently found to be downregulated in various other tumors, such as chondrosarcoma, gastric cancer and breast cancer [54–56]. There is a positive feedback of K-RAS expression system: the PI3K/AKT and MAPK/ERK1/2 pathways downstream of K-RAS can also promote the transcription of K-RAS. MiRNAs have been considered as potential targets for tumor therapy, but they are easily degraded by RNA nucleases. Therefore, efficient miRNA modification is necessary. Yukihiro et al. enhanced the resistance of miR-143 to nucleases by altering the sequence of the passenger strand in the miR-143 duplex and modifying the 3′-overhang region of double-stranded miR-143 [57]. This chemically modified miR-143 interferes with the expression system and activation of K-RAS by silencing Sos1, AKT, and ERK, and its combination with low-dose EGFR inhibitors can significantly inactivate the MAPK/ERK and PI3K/AKT pathways, restoring the effect of EGFR inhibitors. Previous therapies that directly target activated K-RAS have been unsuccessful in clinical applications, and the oncoprotein RAS is therefore considered an "untreatable" target for cancer. MiR-143 gives us a better understanding of the K-RAS signaling network and demonstrates the possibility of developing RNA drugs for K-RAS-driven cancers [58]. MiR-145-5p inhibits the EGFR/RAF/MEK/ERK pathway by targeting rhomboid domain containing protein 1 (RHBDD1) and its downregulation in CRC is associated with poor prognosis [59]. KRAS and Ras-responsive element binding protein 1 (RREB1) are targets of miR-143 and miR-145. In addition to downregulating a set of genes in the MAPK signaling cascade, miR-143 and miR-145 also abolish signaling through the MAPK, PI3K, and JNK pathways by downregulating KRAS and RREB1 [60]. As a downstream transcription factor of the RAS/MAPK pathway, alterations of c-MYC may be involved in mCRC resistance to anti-EGFR mAbs. MiR-143 and miR-145 are negative regulators of c-MYC [61], and their overexpression can increase Cetuximab sensitivity by enhancing the antibody-dependent cellular cytotoxicity (ADCC) [62].

MiR-302a is encoded by the 4q25 region of human chromosome 4 [63], and plays a tumor suppressor role in the occurrence and development of various malignant tumors such as CRC, gastric cancer, and breast cancer. Its downregulation is associated with drug resistance and poor prognosis in multiple tumors [64]. MiR-302a is downregulated in Cetuximab-resistant CRC cells. CRC patients with low levels of miR-302a generally have poorer OS. Mechanistically, miR-302a inhibits CRC metastasis by directly targeting nuclear factor IB (NFIB), a transcriptional activator of integrin subunit alpha 6 (ITGA6). CD44 is another negative regulatory target of miR-302a. Overexpression of miR-302a can block CD44-mediated EGFR/RAS/RAF/MAPK and EGFR/PI3K/AKT signaling pathways and the cancer stem cells (CSC)-like properties in a dose-dependent manner, thereby restoring CRC sensitivity to Cetuximab [65].

Compared with wild-type, miR-378 expression is generally downregulated in CRCs harboring mutations in the KRAS and BRAF genes. Downregulation of miR-378 may be implicated in Cetuximab resistance through the RAS/RAF/MAPK and ERK pathways [66, 67]. Moreover, miR-378 directly targets BRAF and regulates the RAS/RAF/MEK/ERK pathway [68].

MiR-193a-3p is downregulated in CRC cells, more significantly in KRAS mutant CRC cells. MiR-193a-3p can directly target KRAS to regulate the expression of the RAS/RAF signaling axis and increase the sensitivity of KRAS mutant and wild-type CRCs to Cetuximab [69]. Overexpression of miR-193a-3p inhibits the reactivation of MAPK signaling and the expression of Mcl1. Mcl1 is the target protein of miR-193a-3p, and inhibiting it can enhance the sensitivity of anti-EGFR mAbs, but does not participate in the reactivation of the MAPK pathway, suggesting that inhibition of Mcl1 partially mediates the increased efficacy of miR-193a-3p to anti-EGFR antibodies [70].

MiR-141-3p is downregulated in CRC. MiR-141-3p regulates the activation of RAS/RAF1/ERK pathway and AKT by inhibiting the expression of EGFR, enhancing the sensitivity of Cetuximab [71].

The Let-7 family is downregulated in CRC and is the most widely studied miRNA for anti-EGFR therapy biomarkers in KRAS-mutated patients. Let-7 can negatively regulate the expression of KRAS as well as other oncogenes such as MYC, IGF1 and HMGA2, which are all key regulators of CRC growth [72]. In KRAS-mutated patients, high levels of Let-7 were significantly associated with better survival under Cetuximab treatment [73], suggesting that Let-7 helps identify patients with KRAS mutations that may still be possible subpopulations that benefit from EGFR inhibitors. In the case of receiving anti-EGFR mAbs treatment, the progression-free survival (PFS) and OS of patients with high Let-7a and Let-7c expression are superior to those with low expression of Let-7a and Let-7c [73, 74]. The researchers considered that Let-7c may modulate the sensitivity of mCRC patients to anti-EGFR drugs by interfering with the primary resistance mechanism of Cetuximab or Panitumumab, and has application potential in screening mCRC patients sensitive to anti-EGFR therapy [47].

MiR-375 expression is reported to be downregulated in CRC, and its downregulation is associated with poorer OS of patients [42, 75–77]. Khondoker et al. showed that miR-375 and Cetuximab have synergistic anticancer effects in CRC, and downregulation of miR-375 can increase the expression of EGFR ligand connective tissue growth factor (CTGF) and enhance CTGF/EGFR/PIK3CA/AKT pathway activation. Moreover, downregulation of miR-375 promotes EGFR/KRAS/BRAF/ERK pathway activation [76]. However, another study found that miR-375 promoted Cetuximab resistance in CRC. PH domain and leucine-rich repeat protein phosphatase 1 (PHLPP1) is a member of the serine/threonine phosphatase family, which negatively regulates the PI3K/AKT and RAF/MEK/ERK signaling pathways by inducing dephosphorylation of AKT at S473 and RAF1 at S338 [78, 79]. MiR-199a-5 and miR-375 are upregulated in Cetuximab-resistant cells, and overexpression of miR-199a-5p and miR-375 can inhibit PHLPP1, thereby enhancing the resistance of CRC cells to Cetuximab, while silence of them has the opposite effect [42]. Since there is no unified conclusion on the role and significance of miR-375 in CRC anti-EGFR mAbs resistance, further studies are needed.

### Bidirectionally regulated miRNAs

Studies have shown that miR-181d is downregulated in CRC and acts as a potential tumor suppressor to inhibit the invasion of CRC cells [80, 81]. The expression of miR-181d is significantly correlated with tumor location, local recurrence and TNM stage. As an independent prognostic factor for CRC, its low expression is associated with poorer OS. MiR-181d can directly target pseudopodium enriched atypical kinase 1 (PEAK1) to regulate MAPK and PI3K/AKT signaling pathways [81]. The regulation of cell invasiveness by MiR-181d is associated with KLK6. There is a regulatory feedback: miR-181d regulates the secretion of KLK6, and its expression decreases when KLK6 is knocked down [80]. However, miR-181d was also found to be upregulated in CRC and involved in the post-transcriptional regulation of c-MYC by targeting FBXL3 and CRY2, as well as in the regulation of KRAS expression [82–85]. One of these studies identified miR-181d as possibly a onco-miRNA in CRC, which seems to be contrary to the above studies [82]. The expression level and significance of miR-181d in different studies are contradictory, which may be related to its complex regulatory mechanism. In addition, factors such as sample size, research methods, and techniques may also influence the results. The expression pattern and clinical value of miR-181d in CRC require further in-depth studies to clarify.

MiR-545 is downregulated in CRC and functions as a tumor suppressor [86]. It can inhibit EGFR expression by affecting the 3′-UTR activity of EGFR mRNA [86], and block the cell cycle by acting on cyclin-dependent kinases 4 (CDK4) [87], thereby inhibiting tumor progression. MiR-545-3p affects CRC progression by acting on Myosin VI (MYO6) [88]. The expression of miR-545-3p is associated with lymph node metastasis, distant metastasis and TNM stage, and its low expression usually indicates poorer OS [89]. However, miR-545 has also been found to be upregulated in CRC, functioning as a tumor promoter [90, 91]. KWOK et al. confirmed that the intron of the host gene FTX could encode miR-545, miR-374b, and miR-421. Among them, miR-545 and miR-374b target retinoic acid-inducible gene I (RIG-I) and PTEN, which are the key antagonists of the PI3K/AKT pathway, respectively, and synergistically promote the PI3K/AKT pathway activation. In contrast, miR-421 can inhibit the expression of miR-374b and miR-545, suggesting that miR-421 has an auto-regulatory effect within the FTX site [90].

### Other miRNAs that may be involved in anti-EGFR resistance

In many studies, the relationship between miRNAs and anti-EGFR resistance has not been thoroughly explored due to the lack of specific resistance experiments. However, since these miRNAs, including miRNA-181d and miR-545, are involved in the regulation of signaling pathways or target genes associated with anti-EGFR resistance and affect tumor malignant biological behavior, we speculate that they may play an important role in regulating the sensitivity of anti-EGFR mAbs. This theory needs to be confirmed by further studies and will be a promising research direction.

MiR-181a is upregulated in CRC tissues [92]. The tumor suppressor WIF-1 is a target of miR-181a. MiR-181a promotes CRC progression and liver metastasis by inhibiting WIF-1, and its overexpression is closely associated with local tumor invasion and lymph node metastasis, which is an independent prognostic factor for poor OS [93]. It has been reported that miR-181a enhances AKT activation by inhibiting PETN expression [94]. However, in a clinical study by Pichler M et al., low miR-181a expression was associated with poorer cancer-specific survival (CSS) and PFS in KRAS wild-type mCRC patients treated with Cetuximab and Panitumumab [95]. Thus, more research is needed to explain this phenomenon.

MiR-31-3p and miR-31-5p are upregulated in CRC. C-MYC is an important transcription factor downstream of the RAS/MAPK pathway and is the key to CRC resistance to EGFR inhibitors. MiR-31-3p directly regulates the expression of c-MYC by directly acting on its negative regulator E2F2, which may affect the resistance to anti-EGFR mAbs. Among patients receiving anti-EGFR therapy, patients with miR-31-3p overexpression have worse PFS than those with lower levels of miR-31-3p expression (5 months *vs.* 9 months) [61, 96]. MiRNA-31-5p activates BRAF and may be implicated in Cetuximab resistance by acting in the downstream signaling pathway of EGFR. High expression of miR-31-5p is associated with shorter PFS in CRC with all wild-type genes [97]. Moreover, miR-31-5p is also implicated in the regulation of the Wnt/β-catenin signaling pathway [98].

MiR-425-5p is upregulated in CRC. MiR-425-5p can target genes related to BRAF/RAS/MAPK, PI3K/AKT, p53 and other pathways [98]. It can also activate the Wnt/β-catenin pathway by targeting CTNND1, promoting EMT [99].

MiR-21 is upregulated in CRC. It has been reported that miR-21 can target to reduce the expression of 15-hydroxyprostaglandin dehydrogenase (15-PGDH) and increase the level of prostaglandin E2 (PGE2), which mediates EGFR activation by releasing EGFR ligands [100, 101]. PTEN is a direct target of miR-21. MiR-21 activates the PI3K/AKT pathway by inhibiting PTEN expression [102–104]. Moreover, upregulation of miR-21 can also lead to the activation of the Wnt/β-catenin and c-MYC [105].

MiR-133b has been proved to function as a tumor suppressor by regulating EGFR in several human cancer types, and it is significantly dysregulated in tumors such as CRC [106], prostate cancer [107] and lung cancer [108]. The downregulation of miR-133b in CRC is associated with aggressive metastasis and shorter survival. EGFR is the direct functional target of miR-133b in CRC cells, and miR-133b targets to inhibit the expression level of EGFR in CRC cells, thereby inhibiting its mediated cell growth and invasion. Compared with miR-133b mimic or Cetuximab alone, combined treatment of miR-133b mimic and Cetuximab had a stronger inhibitory effect on CRC cell growth and invasion. These results suggest that mCRC patients may benefit from miR-133b combined with Cetuximab therapy [106].

MiR-320d is one of the members of the miR-320 family, and serum miR-320d is considered to be a promising non-invasive diagnostic biomarker for distinguishing metastatic from non-metastatic CRC [109]. Circulating miR-320d is significantly associated with tumor size, lymph node metastasis and distant metastasis [110]. MiR-320d is significantly downregulated in CRC cells and tissues, and can directly negatively regulate the expression of tumor suppressor candidate gene 3 (TUSC3), thereby inhibiting malignant biological behaviors of tumors such as proliferation, migration, invasion and EMT. Moreover, miR-320d can inhibit the PI3K/AKT/mTOR pathway and its mediated pathophysiological effects in CRC [111].

MiR-192, located on chromosome 11, is considered a metastasis suppressor in various cancers, and its expression is downregulated in CRC [112]. MiR-192 can interact with the 3′-UTR of thyroid hormone receptor interactor 13 (TRIP13) mRNA and negatively regulate the expression of TRIP13 at the post-transcriptional level. TRIP13 is involved in regulating the Wnt/β-catenin pathway and activates EGFR/AKT signaling by interacting with fibroblast growth factor receptor 4 (FGFR4) [113]. Moreover, miR-192 can also target APC, c-Fos, etc*.* [114].

The expression of miR-802 is significantly downregulated in CRC cells and tissues, which is related to poor differentiation, higher T, N and TNM stage, as well as poorer OS [115]. The small GTPase Ran can regulate EGFR expression and ERK and AKT pathways activation. MiR-802 is an upstream regulator of Ran and directly targets the 3′-UTR of Ran to downregulate its expression [116].

MiR-497 is downregulated in CRC, and it inhibits PI3K/AKT and MAPK pathway activation by inhibiting insulin receptor substrate 1 (IRS1) [117]. In addition, IGF1R is another target gene of miR-497, and inhibition of IGF1R by miR-497 also downregulates PI3K/AKT pathway activation [118]. Moreover, miR-497 is adsorbed by the lncRNA TTN-AS1, both of which act together in the PI3K/AKT/mTOR signaling pathway [119].

MiR-4689 is significantly downregulated in CRC, especially in KRAS-mutated CRC. MiR-4689 inhibits the activation of RAF/MEK/MAPK, RAF/MEK/ERK and PI3K/AKT pathways by targeting KRAS and AKT1 [120], suggesting that miR-4689 provides a new possibility for targeted therapy of refractory KRAS mutant CRC.

MiR-130a-3p targets Wnt family member 1 (WNT1) to inhibit the expression of its downstream factors c-MYC and cyclin D1. It has been confirmed to be downregulated in CRC [121].

MiR-708-5p is downregulated in CRC and implicated in multiple pathways of anti-EGFR resistance. Upregulation of CD44 and the isoforms can enhance the activation of EGFR signaling pathways [65]. CD44 is the target of miR-708-5p, and downregulation of miR-708-5p can increase CD44 expression and EGFR pathway activation [122]. In addition, miR-708-5p can inhibit the Wnt/β-catenin pathway activation by targeting CTNNB1 [123]. In colorectal adenocarcinoma, miR-708-5p is also implicated in the regulation of the PI3K/AKT pathway [124]. Furthermore, lncRNAs LOXL1-AS1 and MINCR are upregulated in CRC, and they may act synergistically with each other as sponges for miR-708-5p [122, 123].

In CRC, miR-520a-3p is downregulated. It can recognize the 3′-UTR binding site of EGFR mRNA directly and mediate post-transcriptional repression of EGFR [125].

MiR-342‐3p is downregulated in CRC [126]. EGFR and BCL-2 are direct targets of miR-342-3p in CRC cells, and their expression is negatively regulated by miR-342-3p. Moreover, lncRNA SCARNA2, overexpressed in CRC cells, acts as a ceRNA to negatively regulate the function of miR-342-3p [127].

The downregulation of miR-215-5p in CRC is associated with lymph node metastasis, later tumor stage and shorter OS in CRC patients. MiR-215-5p regulates the EGFR signaling pathway by regulating the EGFR ligand epiregulin (EREG) and its transcriptional inducer HOXB9 [128]. In a study comparing the relationship between miR-215-5p expression level and clinical prognosis, the 5-year OS rate of the patients with miR-215-5p high expression was superior to that of the low expression group (53.57% vs. 40.91%) [129].

MiR-99a is downregulated in CRC with a potential target gene: mTOR. Overexpression of miR-99a can negatively regulate the expression of mTOR by binding to the 3′-UTR of mTOR mRNA [130]. In patients treated with anti-EGFR mAbs, those with high miR-99a expression have longer PFS (6.5 *vs.* 2.1 months, *P* = 0.005, HR = 0.36) and OS (24.9 *vs.* 8.4 months, *P* = 0.03, HR = 0.34) than those with low miR-99a expression [47].

Eph receptors comprise a family of receptor tyrosine kinases. Studies have shown that there is crosstalk between the EphA2 and EGFR pathways, the key targets of MAPK activity are located downstream of EGFR and EphA2 receptors, and EphA2 and EGFR overexpression has been shown a combined effect associated with Cetuximab resistance independent of KRAS mutation status [131–133]. MiR-200a and miR-26b were downregulated in CRC, which is associated with poor prognosis. They targeted both EphA2 and EGFR pathways, and negatively correlated with their target protein expression. It is considered that miR-200a and miR-26b participate in the regulation of the EphA2/EGFR/MAPK pathway by regulating the expression of EphA2 and EGFR [131].

MiR-875-5p is another miRNA that significantly downregulated in CRC. Zhang et al*.* first confirmed that the oncogene EGFR is the target of miR-875-5p. MiR-875-5p negatively regulates EGFR expression by targeting the 3′-UTR of EGFR mRNA. They also found that reduced miR-875-5p expression was related to poor prognosis in CRC [134].

## LncRNAs and anti-EGFR therapy resistance

### Overview of lncRNAs

LncRNAs are enriched in cytoplasmic [135], and can be broadly classified into cis-transcripts, which regulate local chromatin structure and/or gene expression, and trans-transcripts, which regulate cellular function [136, 137]. LncRNAs regulate gene expression at the pre-transcriptional level through gene modification, chromatin remodeling or histone modification [138]; They regulate gene transcription by interacting with transcription factors during transcription [139]; They also regulate gene expression as precursors of some miRNAs, or regulate the translation of mRNAs as ceRNAs at the post-transcriptional level [140]. Initially, lncRNAs were considered as "junk" transcripts with no function, but subsequent studies have confirmed that lncRNAs are widely implicated in various physiological processes, as well as the occurrence and development of some diseases, such as cancer, cardiovascular diseases, autoimmune diseases, and diabetes [26, 141–143]. Studies have shown that lncRNAs precisely regulate the tumor initiation, progression, recurrence and metastasis, and the relationship between lncRNAs and tumor resistance to therapy is received increasing attention due to its complex interaction network [144–147].

## Upregulated lncRNAs

MIR100HG, encoded by the MIR100HG gene on chromosome 11 [148], is upregulated in Cetuximab-resistant CRC cells [41]. Liu et al*.* [44] found that MIR100HG and heterogeneous nuclear ribonucleoprotein A2B1 (hnRNPA2B1) synergistically enhance the stability of TCF7L2 mRNA, a downstream effector of Wnt/β-catenin signaling, in an m6A-dependent manner, regulate the transcriptional activity of Wnt/β-catenin signaling pathway and promote Cetuximab resistance. Not only MIR100HG and hnRNPA2B1 can synergistically enhance the stability of TCF7L2 mRNA, but TCF7L2 can also activate MIR100HG transcription, forming a positive feedback regulatory loop. Based on previous research results, miR-100 and miR-125b synergistically promote Cetuximab resistance by targeting several negative regulators of Wnt signaling [41]. Therefore, a complementary mechanism for enhancing Wnt/β-catenin signaling in mCRC is proposed: MIR100HG and miR-100, miR-125b act at different levels of this signaling pathway to promote Cetuximab resistance.

UCA1 acts as a ceRNA to regulate the target genes expression at the post-transcriptional level [149–151], and it is overexpressed in CRC cells and tissues. As a sponge for miR-495, UCA1 binds and inhibits miR-495, promotes the expression of hepatocyte growth factor (HGF) and c-MET, activates the HGF/c-MET signaling pathway in CRC cells, and attenuates the Cetuximab-induced cell proliferation inhibition, thereby promoting Cetuximab resistance [32].

CRART16 is a ceRNA highly expressed in Cetuximab-resistant CRC cells. CRART16 is a sponge of miR-371a-5p. By negatively regulating miR-371a-5p, it increases the expression of target gene ERBB3, activates the ERBB3/MAPK pathway, and promotes Cetuximab resistance. Moreover, CRART16 overexpression can enhance CSC transformation, which in turn promotes Cetuximab resistance [33]. CRART16 can serve as both a marker for predicting Cetuximab sensitivity and a potential therapeutic target for Cetuximab resistance.

The expression of LINC00973 is significantly upregulated in Cetuximab-resistant CRC cells. Inhibition of LINC00973 can improve Cetuximab resistance. The specific mechanism is not clear, and it may be related to glucose metabolism. Knockdown of LINC00973 can improve cell resistance to Cetuximab. LINC00973 expression level can be used as a factor predicting Cetuximab resistance [152].

SLCO4A1-AS1 is highly expressed in CRC cells and tissues and is associated with poor prognosis. SLCO4A1-AS1 can activate the EGFR/MAPK pathway [153], and also inhibit the degradation of β-catenin and increase the activation of the Wnt/β-catenin signaling pathway [154]. Moreover, SLCO4A1-AS1 has been reported to increase the level of CDK2 by enhancing the interaction between CDK2 and heat-shock protein 90 (HSP90), activating the c-MYC signaling pathway [155].

TTN-AS1, a newly discovered lncRNA located in the 2q31.2 region of human chromosomes, is significantly increased in CRC tissues and cells. TTN-AS1 overexpression predicts poor prognosis for CRC patients. TTN-AS1 is a sponge of miR-497, and its overexpression activates PI3K/Akt/mTOR signaling in CRC cells, partially mediated by regulating miR-497 [119].

### Downregulated lncRNAs

POU5F1P4, also known as OCT4-pg4, located on chromosome 1, is a pseudogene for octamer-binding transcription factor 4 (OCT4). POU5F1P4 is transcribed from an exon structure similar to OCT4A, but does not produce a stable protein [156]. Peng et al*.* found that POU5F1P4 is downregulated in CRC, and its downregulation may enhance primary and acquired resistance to Cetuximab through the PI3K/AKT pathway [157]. Based on the Affymetrix HGU133A 2.0 array, they found that 9 lncRNAs, including POU5F1P4, are differentially expressed between disease controls and non-responders, of which 5 lncRNAs are significantly associated with PFS in patients, and patients with high expression have significantly prolonged PFS. Subsequently, they validated in clinical samples that downregulation of POU5F1P4 promotes mCRC resistance to Cetuximab. Through GO and KEGG analyses, they speculated that the induction of Cetuximab resistance by POU5F1P4 downregulation may be related to the PI3K/AKT signaling pathway.

### Other lncRNAs that may be involved in anti-EGFR resistance

There are some lncRNAs whose relationship to the sensitivity of CRC patients to anti-EGFR therapy has not been confirmed by clinical trials, but these lncRNAs can act on the signaling pathways that are definitely related to anti-EGFR resistance. Some of them can form ceRNA networks with miRNAs and their tumor-associated target genes, thereby affecting CRC growth and invasion. Therefore, we speculate that they may be associated with anti-EGFR mAbs resistance and are potential therapeutic targets and predictive biomarkers for CRC patients.

Aberrant glycosylation catalyzed by specific fucosyltransferases (FUTs) is associated with malignant behavior of tumors. HOTAIR and FUT6 are upregulated in CRC, and overexpression of HOTAIR is associated with poor clinical prognosis. MiR-326 is downregulated in CRC, HOTAIR is a sponge of miR-326, and there is a negative effect between them. FUT6 is a target gene regulated by miR-326, and the HOTAIR/miR-326/FUT6 axis triggers the PI3K/AKT/mTOR pathway by modifying CD44-α1, 3 glycosylation [158]. Moreover, HOTAIR is also implicated in the regulation of EGFR as a sponge of miR-545 [86].

The expression of ZEB1-AS1 is significantly upregulated in CRC, which is associated with poor prognosis in patients with colon adenocarcinoma [159]. ZEB1-AS1 is a sponge of miR-181a-5p and negatively regulates miR-181a-5p. It regulates the Wnt/β-catenin signaling pathway by deactivating the inhibition of miR-181a-5p on its target β-catenin. In addition, ZEB1-AS1 also affects the expression of downstream target genes TCF4, cyclin D1, MYC, and MMP-7, all of which are associated with proliferation and apoptosis [160]. Similar to ZEB1-AS1, CRNDE is upregulated in CRC and can also regulate the Wnt/β-catenin signaling by adsorbing miR-181a-5p and eliminating its inhibition on the target β-catenin and TCF4 [161].

DNAJC3-AS1 is overexpressed in CRC and is correlated with local invasion, later clinical stage, and poor survival of CRC patients. It is confirmed that DNAJC3-AS1 can induce the EGFR/PI3K/AKT signaling pathway activation [162].

The expression of LINC00265 is increased in CRC cells and tissues, which is associated with lymph node metastasis and later pathological stage. LINC00265 enhances the Wnt/β-catenin signaling and targets EGFR to induce CRC cell proliferation, migration, and apoptosis inhibition [163]. The expression level of LINC00265 correlates with the prognosis of patients: the OS of patients with high LINC00265 expression is inferior to that of patients with lower expression [164].

LOXL1-AS1 is upregulated in CRC. By adsorbing miR-708-5p, it increases the expression of the target gene CD44, which in turn promotes EGFR expression [122]. CTNNB1 is another target gene of miR-708-5p, encoding β-catenin. As an upregulated lncRNA in colon cancer, MINCR promotes the expression of CTNNB1 and increases the activation of Wnt/β-catenin signaling pathway by adsorbing miR-708-5p [123].

SCARNA2 is also upregulated in CRC and is associated with poor prognosis. Overexpression of SCARNA2 competitively binds miR-342-3p and promotes the expression of the target genes EGFR and BCL2 [127].

## CircRNAs associated with anti-EGFR therapy resistance

### Overview of circRNAs

CircRNAs, a newly discovered class of ncRNAs, are considered small endogenous RNAs, and they are widely distributed and rich in variety and have multiple regulatory functions [165]. CircRNAs can be classified into three categories according to their origin: exonic circRNAs (EciRNAs), exon–intron circRNAs (EIciRNAs), and circular intronic RNAs (ciRNAs). CiRNAs and EIciRNAs are restricted to the nucleus and are involved in regulating the transcription of parental genes; EciRNAs are mostly located in the cytoplasm and can act as miRNA sponges in post-transcriptional regulation [165–167]. In addition, a small fraction of circRNAs can be translated into proteins [168]. Generally, circRNAs are present at lower levels compared to the corresponding linear RNAs [169]. Unlike linear RNAs, circRNAs are single-stranded, covalently closed circular transcripts without 5′ caps and 3′ tails. Therefore, circRNAs are considered more stable than linear RNAs and not easily degraded by RNA exonuclease and have longer half-lives. In most species, the half-life of EciRNAs in cells exceeds 48 h, while the average half-life of mRNA is 10 h [170]. This conserved nature of circRNAs has attracted much attention in recent years, and it has been speculated that circRNAs have potential as therapeutic targets. With the development of research, it has been found that dysregulated circRNAs are involved in the progression of various human diseases, such as diabetes, neurological diseases, cardiovascular diseases, and tumors [52, 171–173]. CircRNAs play an important role in tumor progression by regulating multiple processes of tumorigenesis and development, such as participating in the regulation of persistent proliferative signals [174], escaping from growth suppressors [175], damaging differentiation signals, promoting proliferation and invasion [176], inducing angiogenesis [177], and regulating tumor microenvironment [178]*.* CircRNAs can act as tumor promoters or suppressors to regulate cancer-related signaling pathways, including the Wnt/β-catenin [179], MAPK/ERK [180] and PTEN/PIK3/AKT [181] signaling pathways*.* Moreover, circRNAs also participate in the regulation of some important molecules such as p53 [182] and K-RAS [178]. Currently, the role of circRNAs in tumor drug resistance has gradually attracted people's attention. For example, knockdown of hsa_circ_0006528 increases breast cancer sensitivity to doxorubicin [180], and downregulation of hsa_circ_0000504 may overcome CRC resistance to 5-FU [183].

CircHIPK3, also known as hsa_circ_0000284, is significantly upregulated in CRC cells and tissues. Inhibiting circHIPK3 expression can reverse the resistance of CRC cells to the EGFR inhibitor Cetuximab. The mechanism may be that circHIPK3 acts as a miRNA sponge to absorb endogenous miR-7 to reduce the activity of miR-7, resulting in increased expression of miR-7-targeted proto-oncogenes (FAK, IGF1R, EGFR, and YY1) [184]. Among them, IGF1R and EGFR can activate PI3K/AKT and MEK/ERK signaling pathways, respectively, to enhance tumor progression and drug resistance [184, 185], and YY1 activates Wnt/β-catenin signaling by promoting CTNNB1 expression to promote tumor growth [186]. Furthermore, the OS of patients with high CircHIPK3 expression is significantly inferior to that of patients with low CircHIPK3 expression, suggesting that CircHIPK3 may be a promising prognostic biomarker for CRC.

CDR1as, also known as ciRS-7, is derived from the cerebellar degeneration-related protein 1 antisense transcript (CDR1AS) and is the most widely studied circRNA. CDR1as is a sponge of miR-7, containing more than 70 conventional binding sites for miR-7 [187]. It is upregulated in CRC and regulates a variety of cellular processes. Since EGFR and IGF1R are the target genes regulated by miR-7, CDR1as/miR-7 axis may promote CRC progression by regulating EGFR and IGF1R. Higher CDR1as expression is associated with poorer OS [52].

CircRNA_0000392 is also overexpressed in CRC cells and tissues. miR-193a-5p can specifically bind to PIK3R3, one of the mammalian class IA PI3Ks genes, and regulate its expression. As a sponge of miR-193a-5p, circRNA_0000392 can negatively regulate miR-193a-5p, relieve its inhibition of PIK3R3, and activate the AKT/mTOR signaling pathway, which may be a potential therapeutic target for CRC [188].

CDR1as and circRNA_0000392 are dysregulated in CRC and specifically act on receptors and target genes closely related to anti-EGFR resistance, which may be important factors affecting the sensitivity of anti-EGFR therapy and deserve further exploration.

## Discussion and prospect

CRC is the second leading cause of cancer-related death worldwide [1], which greatly threatens human life and health. Since the advent of anti-EGFR mAbs, the prognosis and quality of life of patients with advanced CRC have been significantly improved. Compared with chemotherapy alone, the combination of Cetuximab or Panitumumab with chemotherapy significantly improves OS in CRC patients, with a median OS exceeding 31 months in the RAS wild-type people [189]. However, the primary and secondary drug resistance to anti-EGFR mAbs limits its application, and exploring the causes and solutions of drug resistance is a major challenge.

**Fig. 3:**
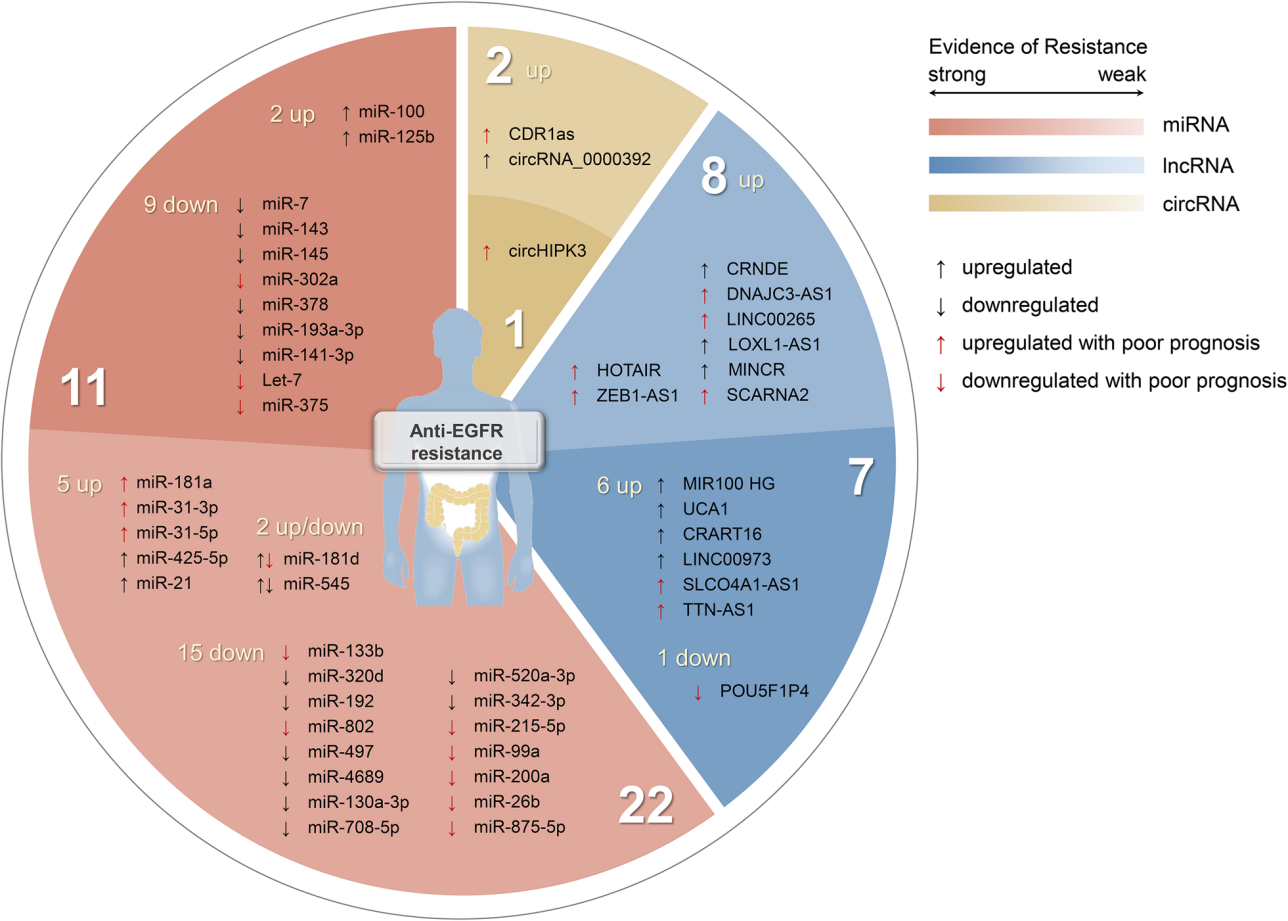
ncRNAs associated with anti-EGFR therapy resistance in CRC. In terms of the number of studies, miRNAs are the most numerous, followed by lncRNAs and circRNAs. The numbers of individual ncRNAs and the strength of the evidence for their association with resistance to anti-EGFR therapy are shown in the figure: ncRNAs in the dark area have been verified by resistance experiments, and ncRNAs in the light area need further verification. ↑ represents upregulated ncRNAs in CRC, ↓ represents downregulated ncRNAs in CRC. ↑ in red represents upregulated ncRNAs is associated with poor prognosis, and ↓ in red represents downregulated ncRNAs is associated with poor prognosis

Currently, studies have shown that inherent genetic mechanisms including KRAS/NRAS/BRAF mutations and ERBB2, MET amplification are the main reasons for the resistance of CRC patients to anti-EGFR therapy, but there are still 30% of cases that do not respond to anti-EGFR therapy from an unknown, apparently non-genetic resistance mechanism [6–8, 11–13]. Recently, epigenetic research on anti-EGFR resistance has gradually become popular. NcRNAs, especially lncRNAs, miRNAs and circRNAs, have been confirmed to be involved in the resistance of anti-EGFR mAbs. Meanwhile, there are complex interactions between these three types of ncRNAs. For example, some lncRNAs can be processed to generate miRNAs that negatively regulate target mRNAs [41], and lncRNAs and circRNAs can act as a ceRNA to competitively bind to miRNAs and eliminate the inhibition of target genes [29, 31]. Chu et al. have reviewed the advance of ncRNAs in anti-EGFR mAbs therapy resistance [190]. In this paper, we conducted a more comprehensive search and update of relevant literature, which not only included more reports of ncRNAs related to anti-EGFR resistance, but also demonstrated the interaction networks between different ncRNAs, providing more detailed and in-depth mechanism exploration and researches on reversal of Cetuximab resistance. In addition, we also introduced some ncRNAs that regulate signaling pathways or genes related to anti-EGFR resistance and affect the malignant biological behavior of CRC, but have not been validated by resistance experiments, which will inspire and help investigators to carry out basic research on the mechanism of anti-EGFR resistance in the future (Tab. 1, Fig. 3). The ceRNA network associated with anti-EGFR resistance is shown in Fig. 2B. In addition to its application in CRC, Cetuximab is also widely used in the treatment of head and neck squamous cell carcinoma (HNSCC). Studies have revealed that ncRNAs are also implicated in Cetuximab resistance in HNSCC. For example, HOTAIR can affect the sensitivity of Cetuximab in HNSCC through the crosstalk between STAT3/HOTAIR/EZH2 and PI3K/AKT signaling pathways [191]. MiR-212 may lead to acquired resistance to Cetuximab by increasing the expression of heparin-binding epidermal growth factor-like growth factor (HB-EGF) and activating receptor kinases other than EGFR [192].

**Table 1:** ncRNAs associated with anti-EGFR therapy resistance in CRC

NcRNAs	Upregulated/downregulated	Target	References
*miRNAs*			
miR-100	Upregulated	DKK1, ZNRF3/Wnt/β-catenin pathway	[41]
miR-125b	Upregulated	ZNRF3, RNF43, DKK3, APC2/Wnt/β-catenin pathway p53 HER2	[41] [46] [47]
miR-7	Downregulated	YY1 TYRO3, PI3K/AKT/mTOR pathway EGFR RAF-1 IGF1R	[49] [50] [51, 52] [51] [52]
miR-143	Downregulated	Sos1, AKT, ERK KRAS, RREB1 MAPK pathway c-MYC ADCC	[58] [60] [60] [61] [62]
miR-145	Downregulated	RHBDD1, EGFR/RAF/MEK/ERK pathway KRAS, RREB1 MAPK pathway c-MYC ADCC	[59] [60] [60] [61] [62]
miR-302a	Downregulated	CD44	[65]
miR-378	Downregulated	RAS/RAF/MAPK pathway ERK pathway BRAF, RAS/RAF/MEK/ERK pathway	[66, 67] [66, 67] [68]
miR-193a-3p	Downregulated	KRAS, RAS/RAF pathway MAPK pathway Mcl1	[69] [70] [70]
miR-141-3p	Downregulated	EGFR, RAS/RAF1/ERK pathway EGFR, AKT pathway	[71] [71]
Let-7	Downregulated	KRAS, MYC, IGF1, HMGA2	[72]
miR-375	Downregulated	CTGF/EGFR/PIK3CA/AKT pathway EGFR/KRAS/BRAF/ERK pathway PHLPP1	[76] [76] [42]
miR-181d	Downregulated	PEAK1 KLK6	[81] [80]
	Upregulated	FBXL3, CRY2 KRAS	[82] [85]
miR-545	Downregulated	EGFR CDK4 MYO6	[86] [87] [88]
	Upregulated	RIG-I, PI3K/AKT pathway	[90]
*Other miRNAs that may be involved in anti-EGFR resistance*			
miR-181a	Upregulated	PTEN WIF-1	[94] [93]
miR-31-3p	Upregulated	E2F2, c-MYC pathway RAS/MAPK pathway	[61, 96] [61]
miR-31-5p	Upregulated	BRAF Wnt/β-catenin pathway	[97] [98]
miR-425-5p	Upregulated	BRAF/RAS/MAPK pathway PI3K/AKT pathway p53 CTNND1, Wnt/β-catenin pathway	[98] [98] [98] [99]
miR-21	Upregulated	15-PGDH, EGFR pathway PTEN, PI3K/AKT pathway Wnt/β-catenin pathway c-MYC	[100, 101] [102–104] [105] [105]
miR-133b	Downregulated	EGFR	[106]
miR-320d	Downregulated	PI3K/AKT/mTOR pathway TUSC3	[111] [111]
miR-192	Downregulated	TRIP13, EGFR/AKT pathway TRIP13, Wnt/β-catenin pathway APC, c-Fos	[113] [113] [114]
miR-802	Downregulated	EGFR, ERK pathway EGFR, AKT pathway	[116] [116]
miR-497	Downregulated	IRS1, PI3K/AKT pathway IRS1, MAPK pathway IGF1R, PI3K/AKT pathway PI3K/AKT/mTOR pathway	[117] [117] [118] [119]
miR-4689	Downregulated	KRAS, RAF/MEK/MAPK pathway KRAS, RAF/MEK/ERK pathway PI3K/AKT1 pathway	[120] [120] [120]
miR-130a-3p	Downregulated	WNT1	[121]
miR-708-5p	Downregulated	CD44, EGFR pathway CTNNB1, Wnt/β-catenin pathway PI3K/AKT pathway	[122] [123] [124]
miR-520a-3p	Downregulated	EGFR	[125]
miR-342-3p	Downregulated	EGFR, BCL2	[127]
miR-215-5p	Downregulated	EREG, HOXB9	[128]
miR-99a	Downregulated	mTOR	[130]
miR-200a	Downregulated	EphA2/EGFR/MAPK pathway	[131]
miR-26b	Downregulated	EphA2/EGFR/MAPK pathway	[131]
miR-875-5p	Downregulated	EGFR	[134]
*lncRNAs*			
MIR100HG	Upregulated	Wnt/β-catenin pathway	[41, 44]
UCA1	Upregulated	HGF/c-MET pathway	[32]
CRART16	Upregulated	ERBB3, ERBB3/MAPK pathway	[33]
LINC00973	Upregulated	/	[152]
SLCO4A1-AS1	Upregulated	EGFR/MAPK pathway Wnt/β-catenin pathway c-MYC pathway	[153] [154] [155]
TTN-AS1	Upregulated	PI3K/AKT/mTOR pathway	[119]
POU5F1P4	Downregulated	PI3K/AKT pathway	[157]
*Other lncRNAs that may be involved in anti-EGFR resistance*			
HOTAIR	Upregulated	EGFR FUT6, PI3K/AKT/mTOR pathway	[86] [158]
ZEB1-AS1	Upregulated	β-catenin, Wnt/β-catenin pathway	[160]
		TCF4, MYC, MMP-7, cyclin D1	[160]
CRNDE	Upregulated	β-catenin, TCF4, Wnt/β-catenin pathway	[161]
DNAJC3-AS1	Upregulated	EGFR/PI3K/AKT pathway	[162]
LINC00265	Upregulated	Wnt/β-catenin pathway EGFR	[163] [163]
LOXL1-AS1	Upregulated	CD44, EGFR pathway	[122]
MINCR	Upregulated	CTNNB1, Wnt/β-catenin pathway	[123]
SCARNA2	Upregulated	EGFR, BCL-2	[127]
*circRNAs*			
circHIPK3	Upregulated	EGFR, MEK/ERK pathway IGF1R, PI3K/AKT pathway YY1, β-catenin pathway	[184] [184] [184, 186]
CDR1as	Upregulated	EGFR, IGF1R	[52]
circRNA_0000392	Upregulated	PIK3R3, AKT/mTOR pathway	[188]

NcRNAs have great application potential in screening potentially beneficial patient groups, predicting the efficacy of anti-EGFR therapy, predicting patient prognosis, as biomarkers, improving drug resistance, and providing new treatment options. For example, Let-7 helps identify a subpopulation of KRAS-mutant patients who may still benefit from anti-EGFR therapy [73]. Patients with high expression of POU5F1P4 have significantly prolonged PFS compared with patients with low expression [157]. MiR-425-5p is used as a marker in blood and stool for non-invasive examination [193]. Combination therapy of miR-133b and Cetuximab enhanced Cetuximab sensitivity [106]. CircHIPK3/miR-7 axis targeting therapy may be a promising therapeutic approach for CRC patients [184]. Moreover, the clinical guiding significance of these ncRNAs can be extended to other types of tumors and targeted drugs, for example, miR-143 or miR-145 overexpression is not limited to increasing Cetuximab-mediated ADCC, but also increasing ADCC mediated by trastuzumab, guiding the treatment of breast cancer [62].

However, the current research on ncRNAs is still limited. The spectrum of ncRNAs associated with anti-EGFR resistance remains largely unknown, mainly due to the lack of ncRNAs-specific microarrays. According to the current research, the influence of ncRNAs on the efficacy of anti-EGFR mAbs is largely mediated by intracellular signaling, but the specific mechanism needs to be further elucidated, and the interaction between ncRNAs and their crosstalk with anti-EGFR resistance-related signaling pathways remains unclear. For example, the mechanism by which the Wnt/β-catenin signaling pathway promotes resistance to anti-EGFR mAbs still needs to be further explored [41]. The roles of some ncRNAs in different studies or different tumors, or even the same tumor may be contradictory, such as the dual function of miR-125b in tumor regulation: it is highly expressed in glioblastoma and myeloid leukemia, acting as a tumor promoter [194, 195], while it is lowly expressed in cutaneous squamous cell carcinoma (CSCC) and hepatocellular carcinoma (HCC), acting as a tumor suppressor [196, 197]. At present, studies on ncRNAs regulating the sensitivity of anti-EGFR therapy are insufficient, and most of them only have small sample sizes, so these results need to be verified by further large-scale studies.

## Conclusion

The relationship between ncRNAs and tumor and drug resistance has become a new research field. This paper briefly reviewed the research progress on the association between ncRNAs and anti-EGFR resistance in CRC, and discussed the relationship between miRNAs, lncRNAs, circRNAs and anti-EGFR mAbs resistance, the possible mechanisms and potential application values. More tumor-related ncRNAs remain to be discovered urgently, and the functions, interaction networks, specific mechanisms of ncRNAs implicated in drug resistance, and their associations with tumor-related signaling pathways need to be further studied. It is believed that with the development of more research and the application of new methods, more ncRNAs related to drug resistance will be discovered and applied to improve treatment sensitivity, patients' survival and quality of life. NcRNAs have broad prospects of application and research value.

## Data Availability

The data supporting the conclusion of this review have been included within the article.

## Abbreviations

EGFR: Epidermal-growth-factor-receptor
mAbs: Monoclonal antibodies
mCRC: Metastatic colorectal cancer
ncRNAs: Non-coding RNAs
lncRNAs: Long non-coding RNAs
miRNAs: MicroRNAs
circRNAs: Circular RNAs
OS: Overall survival
NCCN: National Comprehensive Cancer Network
snRNAs: Small nuclear RNAs
snoRNAs: Small nucleolar RNAs
piRNAs: PIWI-interacting RNAs
siRNAs: Small interfering RNAs
ceRNAs: Competitive endogenous RNAs
3′-UTR: 3′ Untranslated region
Ago: Argonaute
miRISC: MiRNA-induced silencing complex
EMT: Epithelial to mesenchymal transition
RISC: RNA induced silencing complex
IGF1R: Insulin-like growth factor-1 receptor
RHBDD1: Rhomboid domain containing protein 1
RREB1: Ras-responsive element binding protein 1
ADCC: Antibody-dependent cellular cytotoxicity
NFIB: Nuclear factor IB
ITGA6: Integrin subunit alpha 6
CSC: Cancer stem cells
PFS: Progression-free survival
CTGF: Connective tissue growth factor
PHLPP: PH domain and leucine rich repeat protein phosphatase
PEAK1: Pseudopodium enriched atypical kinase 1
CDK4: Cyclin-dependent kinases 4
MYO6: Myosin VI
RIG-I: Retinoic acid-inducible gene I
CSS: Cancer-specific survival
15-PGDH: 15-Hydroxyprostaglandin dehydrogenase
PGE2: Prostaglandin E2
TUSC3: Tumor suppressor candidate gene 3
TRIP13: Thyroid hormone receptor interactor 13
FGFR4: Fibroblast growth factor receptor 4
IRS1: Insulin receptor substrate 1
WNT1: Wnt family member 1
EREG: EGFR ligand epiregulin
hnRNPA2B1: Heterogeneous nuclear ribonucleoprotein A2B1
HGF: Hepatocyte growth factor
HSP90: Heat-shock protein 90
OCT4: Octamer-binding transcription factor 4
FUTs: Fucosyltransferases
EciRNAs: Exonic circRNAs
EIciRNAs: Exon–intron circRNAs
ciRNAs: Circular intronic RNAs
CDR1AS: Cerebellar degeneration-related protein 1 antisense transcript
HNSCC: Head and neck squamous cell carcinoma
HB-EGF: Heparin-binding epidermal growth factor-like growth factor
CSCC: Cutaneous squamous cell carcinoma
HCC: Hepatocellular carcinoma
MREs: MiRNA response elements
